# High Expression of CKS2 Predicts Adverse Outcomes: A Potential Therapeutic Target for Glioma

**DOI:** 10.3389/fimmu.2022.881453

**Published:** 2022-05-19

**Authors:** Kai Yu, Yulong Ji, Min Liu, Fugeng Shen, Xiaoxing Xiong, Lijuan Gu, Tianzhu Lu, Yingze Ye, Shi Feng, Jianying He

**Affiliations:** ^1^ Department of Neurosurgery, Renmin Hospital of Wuhan University, Wuhan, China; ^2^ Key Laboratory of Translational Cancer Research, Jiangxi Cancer Hospital of Nangchang University, Nanchang, China; ^3^ Department of Neurosurgery, Poyang County People’s Hospital, Shangrao, China; ^4^ Bone Traumatology Department, Shangli County Traditional Chinese Medicine Hospital, Pingxiang, China; ^5^ Central Laboratory, Renmin Hospital of Wuhan University, Wuhan, China; ^6^ Department of Orthopedic, JiangXi Provincial People’s Hospital, The First Affiliated Hospital of Nanchang Medical College, Nanchang, China

**Keywords:** glioma, cyclin-dependent kinases, bioinformatics, biomarkers, prognosis, immune related

## Abstract

Cyclin-dependent kinase regulatory subunit 2 (CKS2) is a potential prognostic marker and is overexpressed in various cancers. This study analyzed sequencing and clinical data from The Cancer Genome Atlas (TCGA) and Gene Expression Omnibus, with external validation using the Chinese Glioma Genome Atlas (CGGA) data. CKS2 expression in the normal brain and tumor tissue was compared. cBioPortal and MethSurv were utilized to scrutinize the prognostic value of CKS2 methylation. Gene set enrichment examination and single-sample gene set enrichment analysis were employed to explore the potential biological functions of CKS2. Cell viability, colony formation, and transwell assays were conducted to evaluate the influence of CKS2 on glioma cell proliferation and invasion. Compared with normal brain tissue, the expression of CKS2 was upregulated in glioma samples (*p* < 0.001). Multivariate data analysis from TCGA and CGGA indicated that increased expression of CKS2 was an independent risk factor for the prognosis of overall survival in glioma patients. CKS2 methylation was negatively associated with CKS2 expression. Patients with CKS2 hypomethylation had worse overall survival compared with patients with CKS2 methylation, as suggested by the analysis of both TCGA and CGGA datasets. The expression level of CKS2 is closely related to tumor immunity, including the correlation of tumor immune cell infiltration, immune score, and co-expression of multiple immune-related genes. In addition, CKS2 is associated with several immune checkpoints and responses to the chemotherapy drug cisplatin. CKS2 knockdown impeded the expansion and aggression of glioma cell lines. The changes in CKS2 expression may provide a novel prognostic biomarker that can be used to improve patient overall survival rates.

## 1 Introduction

Glioma is the most typical malignant brain cancer (1), with approximately 10,000 new cases each year (2). In 2016, WHO defined grades II and III gliomas as diffuse lower-grade gliomas (LGG), with a 5-year survival rate of approximately 50% and 30%, respectively (3). Grade IV gliomas were defined as glioblastoma (GBM), with a median overall survival (OS) of 12–14 months, a 5-year survival rate of only 9%, and the worst prognosis (4, 5). Gliomas are distinguished characterized by fast development, heightened infiltration, and difficult surgical resection. Most patients with glioma are diagnosed at grade IV (6). For decades, histology has been the gold standard for the classification and evaluation of glioma prognosis, as well as for disease management (7). Exhaustive genomic analysis of low- and high-grade gliomas has documented that the heterogeneity of glioma tumors increases with tumor grade and therapy antagonism, highlighting the need for a better understanding of their underlying biology.

Gliomas originate from diverse glial cells, including astrocytes, oligodendrocytes, and ependymal cells (8). These highly heterogeneous cells may contain numerous subregions with different genotypes, accumulation possibility, invasiveness, hypoxia levels, and therapeutic resistance (9). Because of the heterogeneity of gliomas, tumor recurrence and drug resistance are inevitable. Thus, it is critical to find novel biomarkers to help clarify the pathological mechanism underlying glioma and develop corresponding treatment strategies.

CKS1 and CKS2 are highly conserved members of the human cyclin-dependent kinase subunit (CKS; also known as CksHs) family, both identified by sequence homology between CKS2 and yeast SUC1 (10–12). CKS1 is needed for SCFSkp2-mediated ubiquitination and degradation of p27Kip1 and the cell cycle G1/S transition (13, 14). Cyclin-dependent kinase regulatory subunit 2 (CKS2) participates in cell cycle regulation (15). It also plays a role in tumor expansion. Numerous reports have demonstrated that CKS2 expression is often elevated in various cancers, including lymphatic cancer (16), bladder cancer (17), breast cancer (18), cervical cancer (19), nasopharyngeal carcinoma (20), melanocytic carcinoma (21), esophageal squamous cell carcinoma (22), hepatocellular carcinoma (23), and Wilms tumor (24). Although CKS2 is overexpressed in various cancers and results in poor prognosis, the specific underlying mechanism and potential role remain unclear, particularly in glioma. Therefore, the prognostic value and potential role of CKS2 in glioma require further investigation.

This study used data from The Cancer Genome Atlas (TCGA) database to comprehensively evaluate the predictive worth of CKS2 mRNA expression, copy number variation (CNV), and methylation status in patients with glioma. We also assessed CKS2 expression in glioma tissues and cell lines. The role of CKS2 in tumor immunity has been evaluated in many aspects, including cellular immune infiltration, co-expression of immune-related genes, immune score, immune checkpoint, and sensitivity to chemotherapy. Cell proliferation and invasion tests enrich the functional role of CKS2 in gliomas. In short, CKS2 may be a new target for immunotherapy in the future.

## 2 Materials and Methods

### 2.1 Data Investment

RNA-seq data from TCGA and The Genotype-Tissue Expression Project (GTEx) were downloaded from UCSC XENA^1^. The data included 1,157 GTEx normal brain controls, 689 glioma tissues, and the relevant clinical information. The Toil software (25) was used to process TCGA, GTEx, and transcription per million reads (TPM) RNA-seq data. On the basis of the median CKS2 expression value, patient data were subdivided into high- and low-expression groups. In addition, The GSE4290 dataset from the Gene Expression Omnibus (GEO) dataset, which included 19 normal brain tissue controls and 65 glioma tissues, was used to verify the differential expression of CKS2. RNA-seq data for mRNAseq_693 (26) and mRNAseq_325 (27) were downloaded from the Chinese Glioma Genome Atlas (CGGA) website^2^, and associated clinical data were used for external validation of results from the survival analysis.

### 2.2 Identification of Differentially Expressed Genes

We implemented the R software package DEseq2 (28) to analyze TCGA_GBM_LGG (glioma) project level 3 HTSeq RNA-seq count data to identify differentially expressed genes (DEGs). The threshold for DEGs was set at |log2 (fold change)| > 2.5 and adjusted *p* < 0.001.

### 2.3 Gene Ontology and Pathway Enrichment Analysis

Gene Ontology (GO) is widely employed in bioinformatics, and the Kyoto Encyclopedia of Genes and Genomes (KEGG) is a resource for comprehending biomolecular exchanges and chemical responses. We conducted GO and KEGG pathway enrichment analysis on DEGs employing the R package’s cluster profile (version 3.14.3) program (29). Genes with *p* < 0.01, a minimum count of 3, and an enrichment factor greater than 1.5 were assessed differentially expressed.

### 2.4 Gene Set Enrichment Analysis

In Gene Set Enrichment Analysis (GSEA), the distribution of genes in a predefined gene set is used to assess their trends in a gene table ordered by phenotypical correlation to establish their role in phenotype definition. Our study used the cluster profile package in R (29) for GSEA. CKS2 mRNA expression was split into the high expression and the overexpression groups to determine the significant difference in function and pathway between the two groups. This analysis used h.all.v7.2.symbols as the reference gene set. The GMT (Hallmarks) in the MSigDB collection was used as the gene set database. We considered significant enrichment when false discovery rate (FDR) < 0.25, *p*-adjust < 0.05, and normalized enrichment score (|NES|) > 1.

### 2.5 Correlation Between CKS2 Expression and Immune Infiltration

Single-sample GSEA (ssGSEA) was conducted with the GSVA package in R (30), and the enrichment scores were calculated using specific gene markers (31) for each type of immune cell to deduce the infiltration of immune cells in each sample. Spearman or Pearson correlation analysis was used to evaluate the relationship between immune cell infiltration and CKS2 mRNA expression. RNA-seq data in the level 3 HTSeq-FPKM format from the LGG-GBM (glioma) project of TCGA were used.

### 2.6 Analysis of CKS2 Methylation Status and Disease Prognosis

CKS2 CNV and methylation data were acquired from cBioPortal (https://www.cbioportal.org/). CKS2 mRNA expression levels in the CKS2 CNV group were compared with those in the CKS2 no CNV group using the Kruskal–Wallis test, whereas the correlation between CKS2 methylation and CKS2 expression was compared using Pearson’s correlation analysis. The SMART web platform^3^ was utilized to compare CKS2 methylation levels in normal and tumor tissues. The prognostic value of CKS2 methylation levels in patients with glioma was explored using MethSurv online tool^4^. CKS2 methylation data for glioma tissue samples were downloaded from CGGA (methyl_159) and used to evaluate methylation levels and prognostic values for different grades of glioma.

### 2.7 Construction and Evaluation of the Prognostic Graph Model

A Cox proportional hazards degeneration model evaluated the affinity of relevant variables with OS. The Akaike information criterion was used to screen variables and incorporate them into the multivariate model. The selected variables were included in the construction of the model. Nomograms were created utilizing the RMS package in R (32). We then calculated the c-index, observed the receiver operating characteristic (ROC) curve, and estimated the validity of the nomogram.

### 2.8 Correlation Between CKS2 and Drug Sensitivity

We collected CKS2-related RNA sequences and clinicopathological and survival data and retained clinical sample information recorded by TGCA. According to the existing database of pharmacogenomics Genomics Cancer Drug Sensitivity (https://www.cancerrxgene.org/), we predicted the CKS2 OS-related reaction to chemotherapy in cancer samples. The 50% maximum inhibitory concentration (IC50) of samples was predicted by ridge regression, and the prediction accuracy was determined using the R package “Prophetic”. Default values were used for all parameters, the “battle” of “allSolidTumours” and the batch effects of the tissue type were removed, and the repeated gene expression was aggregated as an average.

### 2.9 Patient Samples

Thirty-four primary glioma samples were collected from an equal number of patients. These underwent surgery, but not chemotherapy or radiotherapy, at Jiangxi Provincial People’s Hospital between September 2020 and May 2021. Normal brain tissue from 10 patients with traumatic or focal epilepsy surgery served as the control. All samples were cryopreserved in liquid nitrogen prior to extraction of total RNA. This examination was supported by the Medical Ethics Committee of Jiangxi Provincial People’s Hospital (No. 2020252037).

### 2.10 Cell Culture

U251 and U87 cell lines were obtained from the American culture collection (Manassas, Virginia, USA). Cells were grown in high glucose DMEM medium supplemented with 10% fetal bovine serum, penicillin-streptomycin mix, and 2 mM glutamine (all from Gibco/Invitrogen Technologies) at 37°C in a humidified incubator with 5% CO_2_.

#### 2.10.1 Cell Transfection

U251 and U87 cells were inoculated in six-well plates and cultivated up to 50%–60% confluency. RiboBio (Guangzhou, China) synthesized small interfering RNA (siRNA) targeting human CKS2. siRNAs were transfected into U251 and U87 cells *via* Lipofectamine^®^ RNAiMAX reagent (Invitrogen, Carlsbad, CA, USA). Transfection reagents and siRNA were diluted with Opti-MEM (Invitrogen) without antibiotics, and subsequent experiments were performed 48 h after transfection.

#### 2.10.2 RNA Extraction and qRT-PCR

Total RNA from tissues or cells was extracted with TRIzol reagent (TaKaRa, Shiga, Japan) and switch transcribed into cDNA using a PrimeScript RT kit (RR047A, TaKaRa). In accordance with the manufacturer’s directions, qRT-PCR in triplicate was conducted using SYBR Premix Ex Taq (Takara, RR820A). Data normalization was performed with GAPDH as a control. Melt curve analysis indicated that a single product was formed in all cases. Relative expression modifications were estimated employing the 2^−ΔΔCt^ method. qRT-PCR primers included convex ring RT primers designed and synthesized by RiboBio. The primers for mRNA CKS2 were 5′-CACTACGAGTACCGGCATGTT-3′ (forward) and 5′-CATGTAATGAACCCAGCCTAGA-3′ (reverse); for GAPDH, 5′-CCCATCACCATCTTCCAGGAG-3′ (forward) and 5′-GTTGTCATGGATGACCTTGGC-3′ (reverse).

#### 2.10.3 Cell Proliferation Assay

Cell viability was determined by Cell Titer 96 Aqueous Reagent (MTS) colorimetric assay (Promega, Madison, WI, USA). In brief, cells (2 × 10^3^ cells per well) were inoculated in 96-well plates 24 h before the experiment. The cells were then divided into three groups (NC, si-CKS2-3, and si-CKS2-4) and incubated for 0, 24, 48, 72, or 96 h. Then, 10 µl of CellTiter 96 Aqueous One Solution Reagent was added to each well and incubated at 37°C for 30 min after the specified time. Absorbance was then measured at 490 nm using a microplate reader (Bio-Rad Laboratories, Inc., Hercules, CA, USA). All experiments were conducted in triplicate.

#### 2.10.4 Colony Formation Assays

U251 and U87 cells were uniformly inoculated in six-well plates with a cell density of 600 cells per well. The cells were transfected with NC, SI-KS2-3, or Si-KS2-4 and cultured in a humidified incubator at 37°C with 5% CO_2_ for 2 weeks. The cells were fixed with 4% paraformaldehyde for 15 min and stained with 0.1% crystal violet (Beyotime Institute of Biotechnology, Shanghai, China) for 20 min. Colonies were photographed using a high-resolution camera (Leica, MC 170 HD) and counted under a microscope.

#### 2.10.5 Cell Invasion Assays

Cell invasion was evaluated with a Transwell chamber (Corning, Inc., Corning, NY, USA). The transfected U251 and U87 cells (2 × 10^4^) were placed in the upper chamber and evenly mixed with a serum-free medium. The lower chamber was loaded with a cell-free medium, including 20% fetal bovine serum. After 48 h of culture at 37°C and 5% CO_2_, the cells were fixed with 4% paraformaldehyde and stained with 0.1% crystal violet. At least five random fields were chosen for cell count under a light microscope, and the ImageJ software was used for analysis.

#### 2.10.6 Statistical Analysis

The Mann–Whitney U test (Wilcoxon rank sum test) was employed to measure differences in CKS2 expression in unpaired samples. Kruskal–Wallis, Mann–Whitney U, and Chi-square tests were utilized to investigate the relationship between CKS2 expression and clinically relevant pathological traits. Univariate and multivariate Cox regression were conducted to establish independent risk factors and construct Cox proportional risk model. The pROC package in R was used to generate ROC curves to evaluate CKS2 expression and the diagnostic performance of nomograms in predicting 1-, 2-, and 5-year OS. Co-expression analysis of CKS2 with immune checkpoints was performed using R package “limma,” “reshape2,” “ggplot2,” “pheatmap,” “immuneeconv,” and “estimate.” The Kaplan–Meier method was utilized to draw a survival curve, and the log-rank test was used to compare the dissimilarity of survival data. We adjusted for potential confounding elements, including sex, age, and clinical stage. All statistical analyses were executed in R v4.0.2 and SPSS 26.0. With a double-tailed test, we set the statistical significance at *p* < 0.05.

## 3 Results

### 3.1 CKS2 Expression Was Upregulated in Gliomas

**Figure 1:**
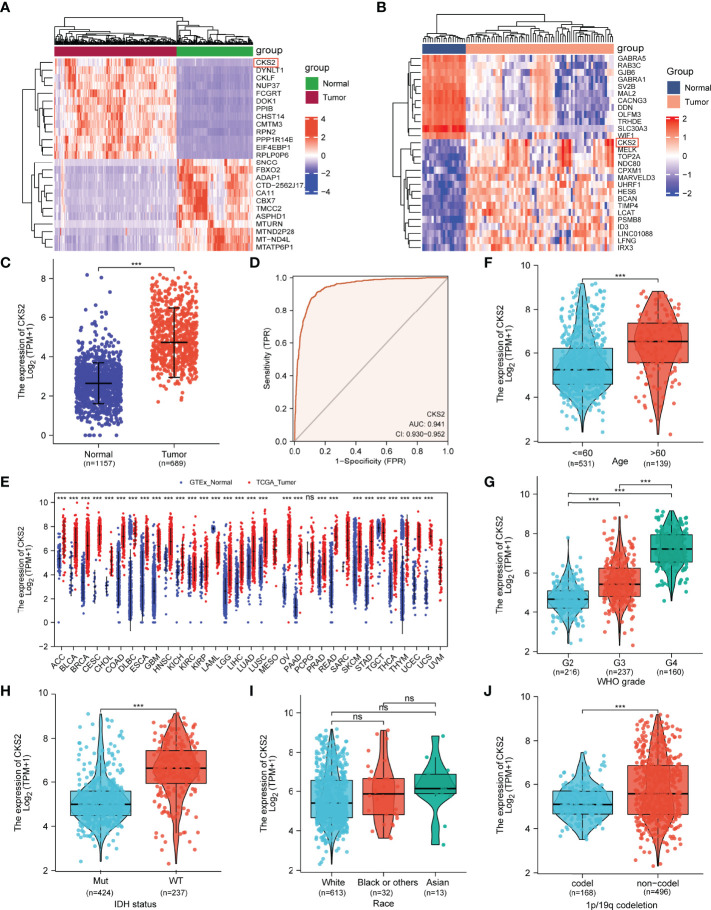
Analysis of CKS2 mRNA expression in glioma and other human cancers in TCGA and GEO databases. **(A, B)** Heatmaps of CKS2 expression levels in the **(A)** GSE4290 and **(B)** TCGA datasets. **(C)** Expression levels of CKS2 in glioma (n = 689) and normal tissue (n= 1157). **(D)** Receiver operating characteristic (ROC) analysis of CKS2 in glioma samples (n = 689). **(E)** CKS2 expression levels in various cancers from TCGA database. **(F)** Association between CKS2 expression and age (n = 613). **(G)** Association between CKS2 expression and histological grade (n = 613). **(H)** Association between CKS2 expression and IDH mutation status (n = 661). **(I)** Association between CKS2 expression and race (n = 613). **(J)** CKS2 expression in 1p/19q co-deletion codel and non-codel (n = 664) (***p < 0.001). NS, not significant.

By comparing data from TCGA (including 104 normal brain tissues and 166 high-grade glioma tissues), GSE4290 (including 22 normal brain tissues and 74 glioma tissues), and combined glioma data from TCGA and GTEx, CKS2 was significantly elevated in glioma tissue compared with normal brain tissue (*p* < 0.001; **Figures 1A–C**). The ROC curve demonstrated that the AUC score was 0.941 (95% CI: 0.930–0.952), and the optimal cutoff value of CKS2 was 3.593 (**Figure 1D**). In RNA-seq data from TCGA, *CKS2* is highly expressed in most cancers (**Figure 1E**), including head and neck squamous cell carcinoma (HNSC), lung adenocarcinoma (LUAD), gastric adenocarcinoma (STAD), cholangiocarcinoma (CHOL), pancreatic adenocarcinoma (PAAD), colon adenocarcinoma (COAD), chromophobia (KICH), adrenal cortical carcinoma (ACC), prostate adenocarcinoma (PRAD), and urothelial bladder carcinoma (BLCA).

CKS2 expression was correlated with patient age, pathological grade, IDH mutation, and 1p/19q chromosome deletion (*p* < 0.001; **Figures 1F–I**). Race and sex were not associated with CKS2 expression (**Figure 1J**). Detailed clinical treatment information is provided in **Table 1**.

**Table 1:** Demographic and clinical characteristics of patients with glioma with low and high expression of CKS2 in TCGA (n = 670).

Characteristic	Levels	CKS2 Expression		*p*
		Low (*n* = 335)	High (*n* = 335)	
Gender (%)	Female	143 (21.3%)	141 (21.0%)	0.938
	Male	192 (28.7%)	194 (29.0%)	
Age [median (IQR)]		39 (31, 49.5)	54 (39, 63)	<0.001
Race	Asian	2 (0.3%)	11 (1.7%)	0.125
	Black or African American	12 (1.8%)	20 (3%)	
	White	315 (47.9%)	298 (45.3%)	
WHO grade	G2	182 (29.7%)	34 (5.5%)	<0.001
	G3	117 (19.1%)	120 (19.6%)	
	G4	6 (1.0%)	154 (25.1%)	
Histological type	Astrocytoma	115 (17.2%)	77 (11.5%)	<0.001
	Glioblastoma	6 (0.9%)	154 (23.0%)	
	Oligoastrocytoma	91 (13.6%)	37 (5.5%)	
	Oligodendroglioma	123 (18.4%)	67 (10.0%)	
IDH status	WT	39 (5.9%)	198 (30%)	<0.001
	Mut	294 (44.5%)	130 (19.7%)	
1p/19q codeletion	codel	109 (16.4%)	59 (8.9%)	<0.001
	non-codel	225 (33.9%)	271 (40.8%)	

TCGA, The Cancer Genome Atlas database; CKS2, cyclin-dependent kinase regulatory subunit 2.

### 3.2 Prognostic Value of CKS2 and Associated Clinical Factors in Glioma

**Figure 2:**
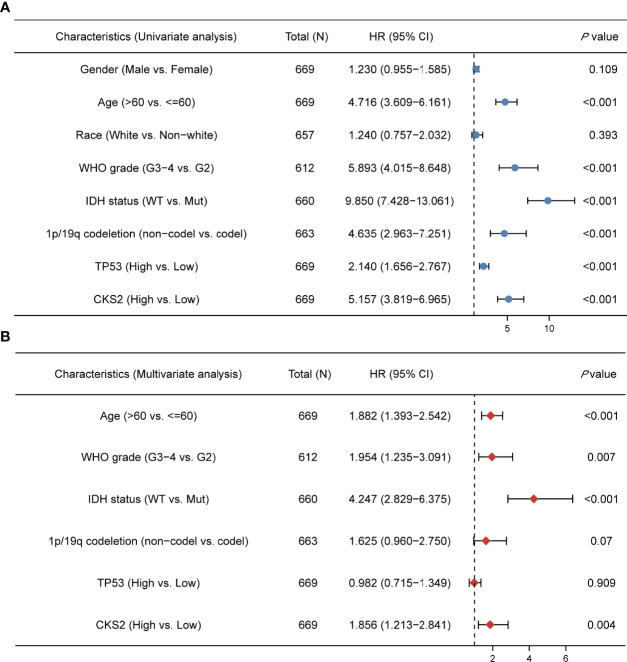
Univariate and multivariate COX regression analysis of CKS2 expression and related clinical factors on overall survival of glioma patients. **(A)** On the basis of the TCGA database, univariate Cox regression showed that CKS2, age, WHO grade, IDH status, TP53, and 1p/19q co-deletion were associated with overall survival (OS). **(B)** Multivariate analysis showed that CKS2 mRNA expression, age, histological grade, WHO grade, and IDH status were independent prognostic factors for OS in glioma.

In all, 386 male and 284 female patients were separated into two groups by age (≤60 or >60 years of age). Glioma tissue samples were categorized into low or high CKS2 mRNA expression groups based on the median TPM value (TPM cutoff of 4.727 was used) (**Table 1**), and univariate and multivariate Cox regression analyses revealed that CKS2 mRNA expression level was a significant independent risk factor for tumor prognosis [hazard ratio (HR) = 1.856; 95% CI: 1.213–2.841, *p* = 0.004]. In addition, age (HR = 1.882; 95% CI: 1.393–2.542, *p* < 0.001), WHO classification (HR = 1.954; 95% CI: 1.235–3.091, *p* = 0.007), and IDH status (HR = 4.247; 95% CI: 2.829–6.375, *p* < 0.001) were an independent prognostic risk factor for OS (**Figure 2**).

External validation was conducted using clinical information for 483 patients in the CGGA dataset** (** **Table S1** **)**. Univariate and multivariate analyses of this dataset also demonstrated that CKS2 mRNA expression was an independent prognostic factor (HR = 1.793; 95% CI: 1.473–2.184, *p* < 0.001) for glioma. WHO grade, IDH status, and 1p/19q co-deletion were also independent factors affecting disease prognosis (**Table 2**). The consistency of results from the external validation dataset increases the reliability of internal queue predictors.

**Table 2:** The univariate and multivariate analyses of overall survival according to CKS2 expression, after adjusting for other potential predictors in CGGA (n = 483).

Characteristics	Total (N)	Univariate Analysis		Multivariate Analysis	
		HR (95% CI)	*P*-value	HR (95% CI)	*P*-value
Gender (Male vs. Female)	483	0.979 (0.817–1.174)	0.823		
Age (>60 vs. <=60)	483	2.125 (1.619–2.789)	<0.001	1.193 (0.901–1.578)	0.218
WHO grade (G3 and G4 vs. G2)	483	4.843 (3.744–6.265)	<0.001	3.069 (2.331–4.040)	<0.001
IDH status (WT vs. Mut)	483	3.116 (2.591–3.748)	<0.001	1.668 (1.362–2.043)	<0.001
1p/19q codeletion (non-codel vs. codel)	483	4.410 (3.256–5.973)	<0.001	2.849 (2.062–3.934)	<0.001
CKS2 (High vs. Low)	483	2.752 (2.279–3.322)	<0.001	1.793 (1.473–2.184)	<0.001

CKS2, Cyclin-dependent kinase regulatory subunit 2.

### 3.3 High CKS2 Expression Was Associated With Adverse Outcomes in Glioma

**Figure 3:**
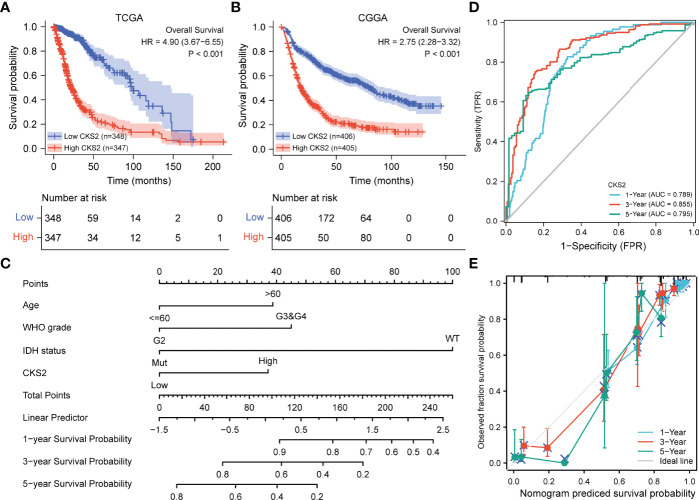
Relationship between the expression of CKS2 in glioma and prognosis. **(A)** Overall survival (OS) curve based on TCGA data (n = 695). **(B)** OS survival curve based on CGGA data (n = 811). **(C)** The nomogram of CKS2 and other glioma prognostic factors from TCGA. **(D)** Time dependent on ROC curve of the line graph. **(E)** Prediction of the calibration curve of the line graph.

Kaplan–Meier survival analysis of the TCGA-LGG-GBM dataset revealed that patients with increased CKS2 expression had a worse prognosis than those with a lower expression (HR = 4.90; 95% CI: 3.67–6.55, *p* < 0.001, **Figure 3A**). To further validate the prognostic value of CKS2 mRNA expression in gliomas, we used RNA-seq data and survival data from 811 patients in the CGGA database. Median CKS2 mRNA expression was 4.66847 based on the Kaplan–Meier median grouping method. Survival analysis demonstrated that patients with high CKS2 expression had poor OS (HR = 2.75; 95% CI: 2.28–3.32, *p* < 0.001, **Figure 3B**). Similarly, we analyzed Kaplan–Meier curves for disease-specific survival (DSS) and platinum-free interval (PFI) in the TCGA-LGG-GBM dataset. Increased CKS2 expression was associated with shorter DSS (HR = 5.17; 95% CI: 3.80–7.03, *p* < 0.001, **Figure S1A**) and PFI (HR = 3.07; 95% CI: 2.45–3.85, *p* < 0.001, **Figure S1B**).

### 3.4 Construction and Evaluation of a Prognosis Model for Glioma

Univariate and multivariate regression analyses identified CKS2 as an independent prognostic factor for glioma. To ascertain this, we established predictive representative maps for OS and DSS based on CKS2 mRNA expression data from TCGA and fitted other clinicopathological parameters. We constructed a nomogram that combined CKS2 expression data with clinical prognostic characteristics, including age, tumor grade, and IDH status (**Figure 3C**). The total score was obtained based on the sum of points allocated to each factor in the graph; the higher the total score, the better the prognosis. Calibration and ROC curves were employed to evaluate the performance of the nomogram. The area under the ROC curve (AUC) values of the nomogram of 1-, 3-, and 5-year OS predicted by CKS2 were 0.789, 0.855, and 0.795, respectively (**Figure 3D**), and the C-index for OS was 0.843 (**Figure 3E**). The higher AUC and C-index determined by the nomograms indicated robust OS predictive discrimination.

### 3.5 Functional Enrichment Analysis of High and Low CKS2-Expressing Samples

**Figure 4:**
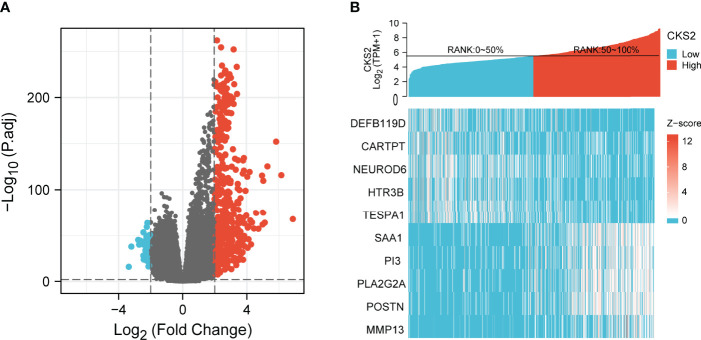
**(A)** The volcano plot of differentially expressed genes (DEGs) was screened according to the expression level of CKS2. **(B)** Heatmap of co-expression with CKS2.

**Figure 5:**
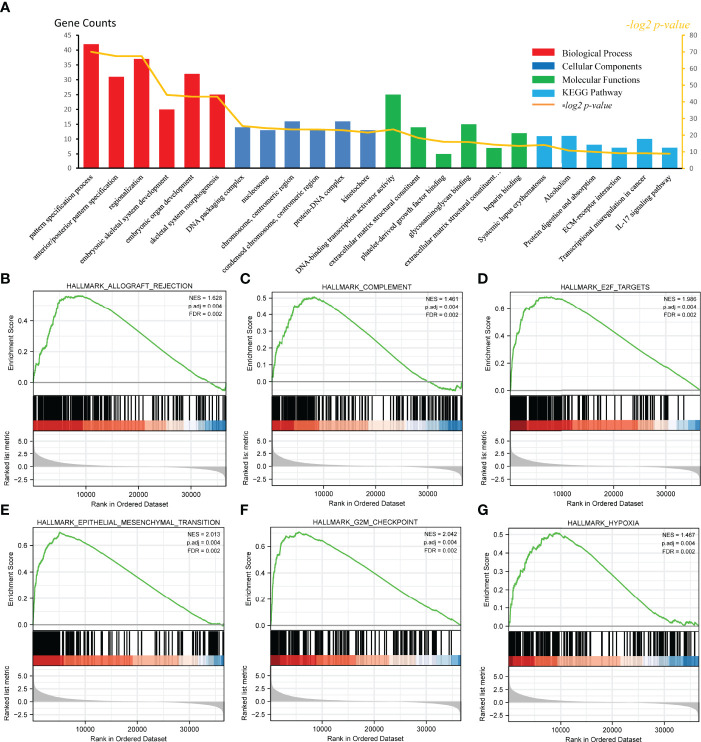
Functional enrichment analysis of CKS2 in glioma. **(A)** Differentially expressed genes (DEGs) were classified as belonging to BP, CC, or MF classes. Gene set enrichment analysis (GSEA) was used to identify enriched gene classes associated with **(B)** allograft rejection, **(C)** complement, **(D)** E2F targets, **(E)** epithelial–mesenchymal transition, **(F)** G2M checkpoint, and **(G)** hypoxia.

To examine the potential mechanism by which CKS2 promotes tumor development, we divided glioma samples into high– and low–CKS2 expression groups. In total, 490 DEGs were identified on the basis of an analysis between the two groups using sequence data from TCGA. Of these, 437 DEGs were highly expressed, whereas expression of 53 was low (selected threshold was |log2 (fold change)| > 2 and *p*-adjust < 0.01, **Figure 4A**). The correlation between CKS2 expression trends and the top 10 genes with *CKS2* co-expression was illustrated using a heatmap (**Figure 4B**). GO enrichment analysis was conducted to predict the functions of co-expression in glioma patients, and enriched terms were ranked on the basis of the adjusted *p*-value. The enrichment terms in biological process (BP), molecular function (MF), cellular component (CC) and KEGG groups were pattern specification process, anterior/posterior pattern specification, regionalization, nucleosome, chromosome, centromeric region, nucleosome assembly, appendage morphogenesis, limb morphogenesis, systemic lupus erythematosus, functional, protein, and absorption (**Figure 5A**). We also conducted GSEA to identify critical pathways associated with CKS2 and found 19 in total (**Table 3**) that met the criteria (FDR < 0.25 and *p* < 0.05). The most significant pathways were allograft rejection (**Figure 5B**), complement (**Figure 5C**), E2F targets (**Figure 5D**), epithelial–mesenchymal transition (**Figure 5E**), G2M checkpoint (**Figure 5F**), and hypoxia (**Figure 5G**).

**Table 3:** Hallmark Pathways enriched in high- and low-risk groups by using GSEA.

ID	NES	P-value	p.adjust	FDR
HALLMARK_ALLOGRAFT_REJECTION	1.628	0.001	0.004	0.002
HALLMARK_COMPLEMENT	1.461	0.001	0.004	0.002
HALLMARK_E2F_TARGETS	1.986	0.001	0.004	0.002
HALLMARK_EPITHELIAL_MESENCHYMAL_TRANSITION	2.013	0.001	0.004	0.002
HALLMARK_G2M_CHECKPOINT	2.042	0.001	0.004	0.002
HALLMARK_HYPOXIA	1.467	0.001	0.004	0.002
HALLMARK_INFLAMMATORY_RESPONSE	1.576	0.001	0.004	0.002
HALLMARK_INTERFERON_GAMMA_RESPONSE	1.763	0.001	0.004	0.002
HALLMARK_MYC_TARGETS_V1	1.470	0.001	0.004	0.002
HALLMARK_TNFA_SIGNALING_VIA_NFKB	1.641	0.001	0.004	0.002
HALLMARK_APOPTOSIS	1.482	0.001	0.004	0.002
HALLMARK_COAGULATION	1.600	0.001	0.004	0.002
HALLMARK_INTERFERON_ALPHA_RESPONSE	1.670	0.001	0.004	0.002
HALLMARK_IL6_JAK_STAT3_SIGNALING	1.710	0.001	0.004	0.002
HALLMARK_ANGIOGENESIS	1.929	0.001	0.004	0.002
HALLMARK_GLYCOLYSIS	1.436	0.002	0.006	0.004
HALLMARK_KRAS_SIGNALING_UP	1.387	0.002	0.006	0.004
HALLMARK_MITOTIC_SPINDLE	1.364	0.003	0.008	0.005

GSEA, gene set enrichment analysis; NES, normalized enrichment score; FDR, false discovery rate.

### 3.6 Hypomethylation Was Associated With *CKS2* Expression and Predicted Adverse Outcomes in Glioma

**Figure 6:**
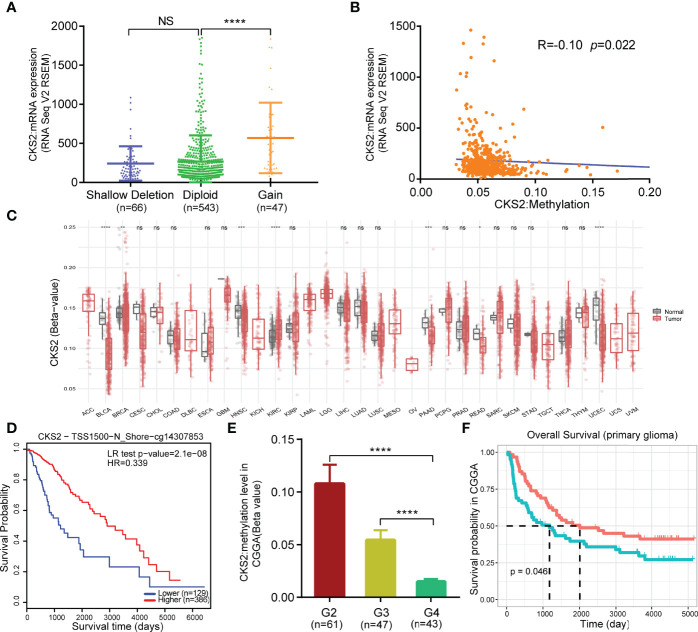
Copy number variation (CNV) and methylation expression of CKS2 in glioma. **(A)** Changes in CKS2 expression levels under different copy numbers (n = 656). **(B)** Correlation between CKS2 methylation level and expression (n = 530). **(C)** Methylation expression levels of CKS2 in various tumor tissues and normal tissues in TCGA data. **(D)** Kaplan–Meier survival of the promoter methylation of CKS2 in glioma (n = 515). **(E)** Association between WHO-defined glioma grade and CKS2 methylation levels in data from the CGGA (n = 151). **(F)** Overall survival curves grouped based on CKS2 methylation levels (n = 123). *p < 0.05, **p < 0.01, ***p < 0.001, and ****p < 0.0001. NS, not significant.

After predicting CKS2 function, we investigated the reasons for the high CKS2 expression. We employed cBioPortal to investigate the association between CKS2 mRNA expression in the CNV and methylation data in glioma samples. No CNV amplification was observed in CKS2, although patients with increased CKS2 CNV had higher CKS2 mRNA expression [7.2% (47/656) of patients, **Figure 6A**]. Copy number variation was not the main factor affecting the high expression of CKS2. We hypothesized that methylation would affect the expression of CKS2 and found that its grade of methylation was negatively associated with mRNA expression (*p* = 0.022, *R* = −0.10, **Figure 6B**). Except for explicit renal cell carcinoma, CKS2 methylation levels were lower in all tumor tissues in TCGA compared with normal tissues, including BLCA, HNSC, PAAD, rectum adenocarcinoma (READ), and UCEC (**Figure 6C**). However, glioma in TCGA lacked normal tissue control samples for comparison. MethSurv analysis revealed that patients with lower CKS2 methylation had poorer OS than those with increased methylation (*p* < 0.001, **Figure 6D**). To further verify the effect of CKS2 methylation levels in glioma, we scrutinized data from the CGGA database and found that higher WHO grades were associated with lower CKS2 methylation (**Figure 6E**). Survival analysis using Kaplan–Meier indicated that patients with lower CKS2 methylation levels had poorer OS compared with patients with higher methylation levels (*p* = 0.046, **Figure 6F**). These results are consistent with those from the experimental group in TCGA dataset.

### 3.7 CKS2 Expression Was Correlated With Immune Cell Infiltration

**Figure 7:**
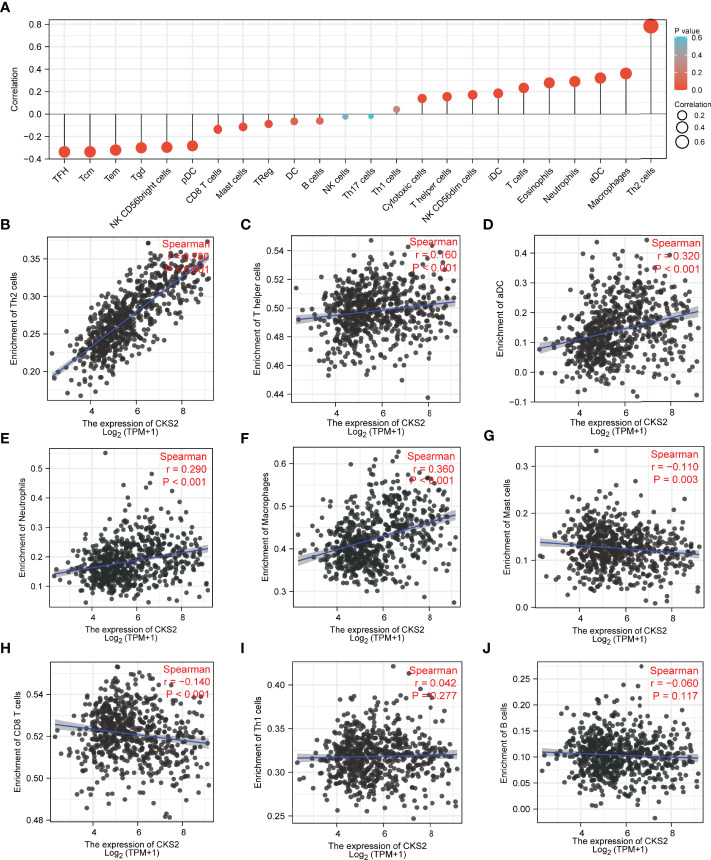
ssGSEA investigation of CKS2 in glioma and correlation between CKS2 expression and immune cell infiltration class. **(A)** Correlation between immune cell infiltration and CKS2 expression. **(B–F)** CKS2 expression and Th2 cells **(B)** and T helper cells (Th) **(C)**. Infiltration levels of activated dendritic cells **(D)**, neutrophils **(E)**, and macrophages **(F)** were positively correlated. **(G, H)** CKS2 expression correlated with the infiltration of mast cells **(G)** and CD8^+^ T cells **(H)** Infiltration levels were negatively correlated. **(I, J)** CKS2 expression was not associated with infiltration levels of Th1 cells **(I)** and B cells **(J)**.

To further explore the role of CKS2 in early-stage glioma, we utilized ssGSEA to research the connection between CKS2 mRNA expression levels and immune cell infiltration. Correlation between the infiltration of 24 immune cells and *CKS2* expression are shown in **Figure 7A**. *CKS2* expression was positively correlated with Th2 cell (*p* < 0.001, *R* = 0.780, **Figure 7B**), helper T cell (*p* < 0.001, *R* = 0.160, **Figure 7C**), activated dendritic cell (*p* < 0.001, *R* = 0.320, **Figure 7D**), macrophage (*p* < 0.001, *R* = 0.360, **Figure 7E**), and neutrophil (*p* < 0.001, *R* = 0.290, **Figure 7F**) infiltration but negatively correlated with mast cell (*p* = 0.001, *R* = −0.110, **Figure 7G**) and CD8^+^ T cell infiltration (*p* < 0.001, *R* = −0.140, **Figure 7H**). ssGSEA analysis also demonstrated that CKS2 mRNA expression was not associated with the infiltration of Th1 (*p* = 0.277, *R* = 0.042, **Figure 7I**) and B cells (*p* = 0.117, *R* = −0.060, **Figure 7J**).

### 3.8 Expression of CKS2 Is Related to Immune-Related Genes, Immune Checkpoints, Immune Score, Sensitivity to Chemotherapy, and Immune Checkpoint Inhibitors

**Figure 8:**
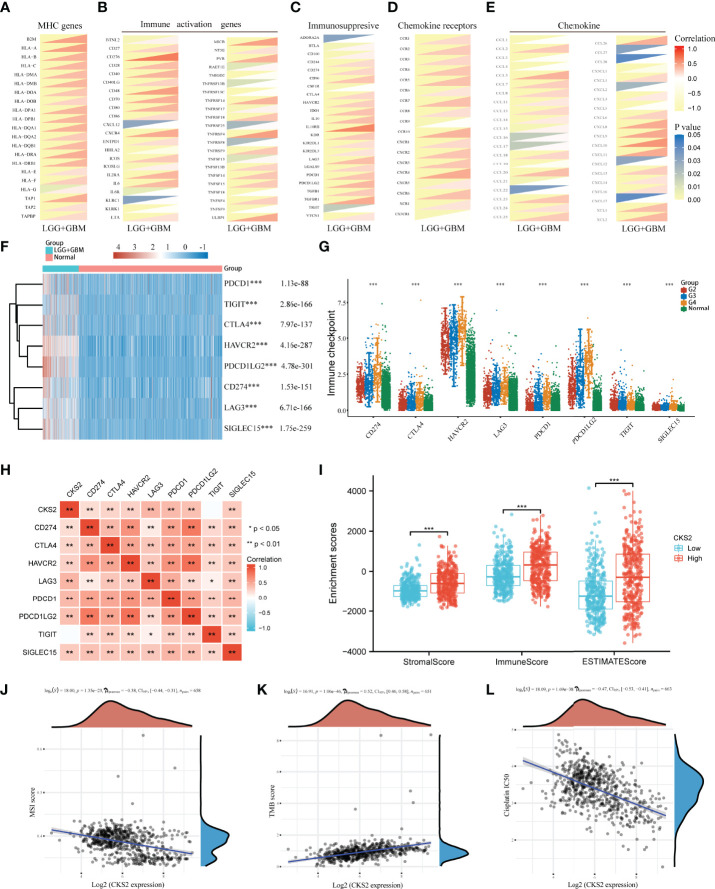
Study on the expression of CKS2 and immune-related genes, immune score, and sensitivity to chemotherapy. CKS2 is co-expressed with **(A)** MHC, **(B)** immune activation, **(C)** immunosuppression, **(D)** chemokine receptors, and **(E)** chemokine genes. **(F, G)** Differential expression of immune checkpoints in normal and LGG_GBM patients. **(H)** Correlation of CKS2 with immune checkpoints in LGG_GBM. **(I)** Correlation of CKS2 with stromal, immune, and estimate scores. **(J, K)** The correlation between CKS2 expression and MSI score and TMB score. **(L)** Correlations CKS2 with the IC50 of chemotherapy drugs. *p < 0.05, **p < 0.01, and ***p < 0.001.

To further clarify the role of CKS2 in glioma immunity, we assessed the co-expression of CKS2 with MHC, immune activation, immunosuppression, chemokine receptors, and chemokines. Among MHC markers, HLA-DMB, HLA-DOB, HLA-E, HLA-F, and TAP2 were co-expressed with CKS2 (**Figure 8A**). CKS2 was significantly correlated with TNFSF14, related to immune activation (**Figure 8B**). Except for ADORA2A, CSF1R, and TIGIT, almost all immunosuppressive genes were positively co-expressed with CKS2 (**Figure 8C**). In addition, CKS2 was highly consistent with CCR4 and CCL25 (chemokine receptors) in terms of chemokine receptors and chemokine markers (**Figures 8D, E** **)**. In addition, we also evaluated the difference in immune checkpoints between glioma and normal groups. It was evident that SIGLEC15, LAG3, and PDCD1 were highly expressed in glioma (**Figures 8F, G** **)**. In addition to TIGIT, through a co-expression heatmap, it can be intuitively found that the expression of CKS2 is positively correlated with immune checkpoint markers (**Figure 8H**). In the immune score analysis, we divided the high and low expression of CKS2 into two groups for ESTIMATE algorithm scoring and found that the expression of CKS2 was significantly negatively correlated with immune score, interstitial score, and ESTIMATE (**Figure 8I**). In addition, both microsatellite instability (MSI) and tumor mutation burden (TMB) play an important role in tumor immunity. CKS2 expression is negatively correlated with the MSI score (**Figure 8J**) and positively correlated with the TMB score (**Figure 8K**). We also evaluated the relationship between CKS2 and a commonly used drug in glioma (cisplatin). The expression of CKS2 was negatively correlated with the IC50 of cisplatin (**Figure 8L**). We further explored the performance of CKS2 in immune checkpoint inhibitors (ICIs). Because transcriptomic data from ICI patients in gliomas are unavailable, we used the Imvigor210CoreBiology dataset (other tumors) to support our guess. The expression of CKS2 in non-responders to ICI [Stable Disease+Progressive Disease (SD+PD)] was lower than that in responders ([Complete Remission+Partial Remission (CR+PR) **Figure S2A**]. Meanwhile, the ORR of low-expression CKS2 was higher than that of high expression CKS2 (71.1% and 83.2%; **Figure S2B**). In addition, according to the high and low expression of CKS2, patients were divided into two groups for survival analysis, and the results showed no significant difference between the two groups (**Figure S2C**).

### 3.9 Knocking Down CKS2 Inhibited the Proliferation and Migration of Glioma Cells

**Figure 9:**
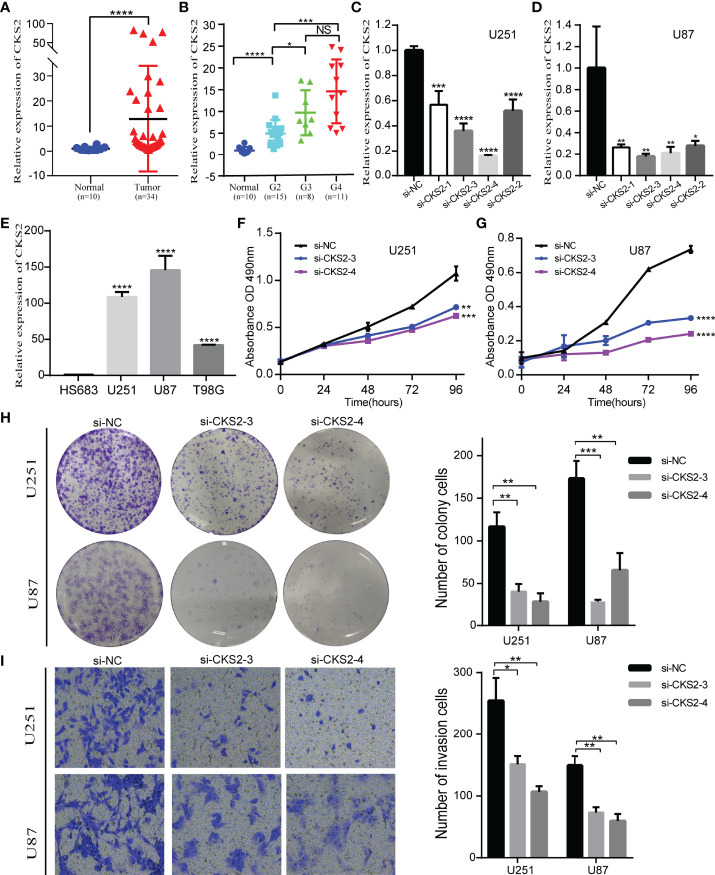
Deletion of CKS2 inhibits the proliferation and invasive abilities of glioma cell lines. **(A)** On the basis of the qRT-PCR analysis, CKS2 expression was particularly upregulated in glioma tissues (n = 34) compared to normal brain tissues (n = 10). **(B) **The association between CKS2 expression and histological grade (n = 34). **(C, D) ** In U251 and U87 cells, the expression of CKS2 was downregulated in the siRNA group. **(E)** The expression of CKS2 in various glioma cell lines. **(F, G)** MTS assay to detect U251 transfected with si-CKS2 and the proliferation inhibition of U87 cells. **(H, I)** Colony formation and transwell assays showed that CKS2 downregulation greatly hindered the proliferation and invasion of U251 and U87 cell lines compared with the control group. *p < 0.05, **p < 0.01, ***p < 0.001, and ****p < 0.0001.

To research the role of CKS2 in glioma, we assessed the effect of CKS2 on glioma cell proliferation and migration. qRT-PCR analysis revealed that CKS2 expression was remarkably upregulated in glioma tissue (n = 34) compared with normal brain tissue (**Figure 9A**). CKS2 expression was also correlated with histological grade (**Figure 9B**). Four siRNAs were employed to quiet CKS2 expression in U251 and U87 cell lines. After transfection and incubation for 48 h, the interference efficiency of siRNA was detected by qRT-PCR. siRNA-CKS2-3 and siRNA-CKS2-4 had the highest silencing efficiency (**Figures 9C, D** **)**. CKS2 was found in four glioma cell lines (HS683, U251, U87, and T98G) and was highly expressed in U251 and U87 cell lines (**Figure 9E**). MTS assay indicated that CKS2 knockout greatly decreased the number of U251 and U87 cells compared with the NC group (**Figures 9F, G** **)**. Colony formation experiments displayed that the deletion of CKS2 remarkably decreased the number of new U251 and U87 cell colonies (**Figure 9H**). In addition, the Transwell assay revealed that CKS2 knockdown reduced the invasion capability of U251 and U87 cells (**Figure 9I**).

## 4 Discussion

Glioma is the prevalent type of intracranial tumor (1). Considering its heterogeneity and poor OS rates (4, 5), evaluating its prognosis effectively and accurately is imperative. In this analysis, we discovered that CKS2 was significantly overexpressed in glioma tissues, and this overexpression was correlated with the hypomethylation of *CKS2*. In addition, high CKS2 expression, high protein expression, and low methylation levels were associated with poor OS. Functional enrichment analysis showed that CKS2 was correlated with allograft rejection, complement, E2F targets, epithelial–mesenchymal transition, G2M checkpoint, and hypoxia. ssGSEA also suggested that high CKS2 expression increased the infiltration of Th2 cells. *In vitro* experiments demonstrated that CKS2 might act as an oncogene in gliomas by affecting cell proliferation and invasion. Therefore, our study offers novel insights into the potential role of CKS2 in tumor pathogenesis and suggests its potential role as a biomarker in glioma.

Data from TCGA and GEO databases indicated that CKS2 is highly expressed in gliomas compared with normal brain tissue (*p* < 0.001). Analysis of patient tissue samples (six normal brain tissues and 37 glioma tissue samples) supported these results. Our analysis of TCGA data confirmed this, suggesting that CKS2 can be a diagnostic marker for a variety of cancers. For glioma, CKS2 expression is a good diagnostic marker, with an AUC as high as 0.941. CKS2 is also associated with the pathological stage of glioma, IDH mutation, 1p/19q co-deletion, and patient age. These clinical data further support the conclusion that CKS2 expression may be related to the degree of glioma malignancy. In addition to its regulatory role in cell cycle transformation, CKS2 also plays a role in tumor development by promoting tumor growth and occurrence (33), promoting proliferation under tumor stress (34), and inhibiting programmed cell death (35). It can also be used as a unique gene marker for malignant cells (36), suggesting that CKS2 may be used as a biomarker for the diagnosis of multiple tumors, including glioma.

CKS2 is highly expressed in gliomas and results in a poor prognosis. Analysis of TCGA-LGG-GBM data showed that patients with high CKS2 expression had worse OS, DSS, and PFI. This was also confirmed using glioma data from the CGGA database. Univariate and multivariate Cox regression revealed that CKS2 was an independent risk factor for glioma. Given that CKS2 is a powerful predictive factor, we assembled a nomogram that combines CKS2 expression with clinical data. With a good C-index and ROC curves, this nomogram predicted the 1-, 3-, and 5-year OS of patients with glioma with high accuracy. This easy-to-use predictive system helps to screen patients with high-risk glioma and identify the best treatment options.

GO term analysis revealed significant enrichment of pathways associated with developmental and cyclical processes such as pattern specification, DNA-binding transcription activator activity, and DNA packaging complex. CKS2 is highly correlated with proliferation and invasion (37, 38). To confirm this in glioma, we conducted MTS, colony formation, and transwell assays. As expected, *in vitro* experiments strongly demonstrated that high CKS2 expression facilitates the proliferation and invasion of glioma cells. Thus, CKS2 may have a role in proliferation and invasion in glioma.

Investigation of the mechanism underlying CKS2 mRNA overexpression indicated that CKS2 hypomethylation was associated with CKS2 overexpression. CKS2 methylation was also associated with poor prognosis in glioma. Contrastingly, ssGSEA showed that CKS2 was closely correlated with immune cell infiltration. CKS2 was positively related to Th2 cell infiltration and negatively correlated with CD8^+^ T-cell infiltration. Earlier studies that have encountered high levels of Th2 cell infiltration and low levels of CD8^+^ T-cell infiltration in various tumors were associated with immunosuppression and low survival (39–41). In this study, Th2 cell levels were particularly elevated, whereas CD8^+^ T-cell levels were reduced, suggesting that CKS2 may help mediate immune escape in glioma (42–44).

TMB is used to assess tumor antigenicity and response to immunotherapy (45). In addition, MSI leads to somatic mutations, potential ICB therapy targets (46). In this study, CKS2 was closely correlated with TMB and MSI, which also explained the vital role CKS2 plays in tumor immunity. Interestingly, CKS2 was also negatively correlated with the IC50 of cisplatin, a commonly used chemotherapy drug. This indicates that patients with glioma with high CKS2 expression may better benefit from ICB treatment. T-cell depletion is a vital link leading to immune dysfunction in tumor patients (47). CKS2 was co-expressed with T-cell depletion markers (PD-1, CTLA4, LAG3, HAVCR2, and GZMB) in this study. Importantly, we also investigated the association between CKS2 and immune checkpoints, in which SIGLEC15, TIGIT, CTLA4, CD274, HAVCR2, LAG3, PDCD1, and PDCD1LG2 were all positively correlated with CKS2, and these markers were also associated with ICB response (48). T-cell depletion and high expression of immune checkpoints predict a worse prognosis in patients, which may explain the cancer-promoting effect of CKS2 in tumors.

Although this study proposes an association between CKS2 and glioma and increases our overall knowledge of the role of CKS2 in glioma, some limitations remain. First, other vital functions and signaling pathways related to glioma may be involved, and these need further investigation. Second, analyzing the role of CKS2 *in vitro* is not sufficient; additional functional studies *in vivo* are needed to corroborate the results of the *in vitro* study.

In conclusion, *CKS2* was overexpressed, whereas CKS2 methylation was decreased in gliomas. In addition, CKS2 knockout inhibited proliferation and invasion of glioma cell lines. Enrichment analysis indicates that CKS2 might be a carcinogenic factor due to its roles in inhibiting immunity, cell cycle regulation, promoting epithelial–mesenchymal transition pathways, and inducing high Th2 and low CD8^+^ T-cell infiltration. CKS2 also plays an important role in tumor immunity and affects the tumor microenvironment, indirectly influencing the prognosis of patients with glioma and paving the way for becoming a potential immunotherapy target in the future. This study demonstrates that CKS2 is a potential marker for glioma diagnosis and prognosis, highlights its role in proliferation and invasion, and shows its potential as an immunotherapy target.

## Data Availability Statement

The original contributions presented in the study are included in the article/**Supplementary Material**. Further inquiries can be directed to the corresponding author.

## Ethics Statement

The studies involving human participants were reviewed and approved by Medical Ethics Committee of Jiangxi Provincial People’s Hospital. The patients/participants provided their written informed consent to participate in this study.

## Author Contributions

KY, XX, and JH jointly participated in the conception and design of the study. Relevant experiments were carried out in YJ, ML, SF, and FS. LG, TL, YY, and JH analyzed the resulting data and edited manuscript drafts. All authors contributed to the article and approved the submitted version.

## Funding

This work was supported by the Health Commission of Jiangxi Provincial (No. 202130032).

## Conflict of Interest

The authors declare that the research was conducted in the absence of any commercial or financial relationships that could be construed as a potential conflict of interest.

## Publisher’s Note

All claims expressed in this article are solely those of the authors and do not necessarily represent those of their affiliated organizations, or those of the publisher, the editors and the reviewers. Any product that may be evaluated in this article, or claim that may be made by its manufacturer, is not guaranteed or endorsed by the publisher.

## Footnotes

1 https://xenabrowser.net/datapages/.

2 http://www.cgga.org.cn/index.jsp.

3 http://www.bioinfo-zs.com/smartapp/.

4 https://biit.cs.ut.ee/methsurv/.

## Abbreviations

TCGA, The Cancer Genome Atlas; CGGA, Chinese Glioma Genome Atlas; OS, overall survival; HR, hazard ratio; LGG, low-grade glioma; GBM, glioblastoma; CNV, copy number variation; GTEx, Genotype-Tissue Expression Project, ROC, receiver operating characteristic.
