# Supplementary material for: High Expression of CKS2 Predicts Adverse Outcomes: A Potential Therapeutic Target for Glioma
Source: Front Immunol. 2022 May 19;13:881453. doi: 10.3389/fimmu.2022.881453 (PMC9160311; doi:10.3389/fimmu.2022.881453)
Supplement: Supplementary file 1 [file DataSheet_1.docx]

Supplementary Material

# Supplementary Figures and Tables

## Supplementary Figures


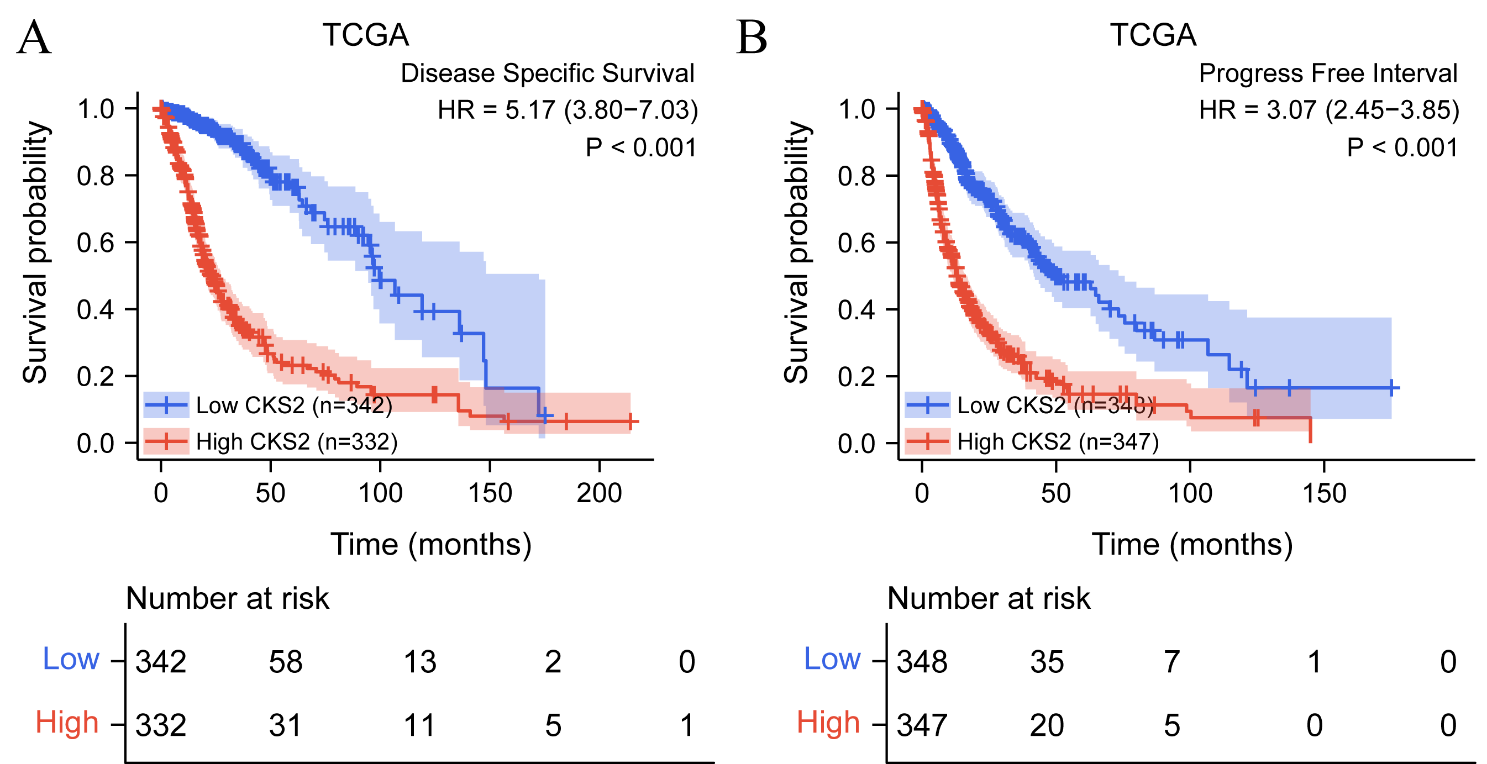


**Supplementary Figure 1. Kaplan-Meier curves for disease-specific survival (DSS) and platinum-free interval (PFI) analysis of TCGA-LGG-GBM dataset.** (**A**) DSS analysis of TCGA data (n = 674); (**B**) PFI survival analysis of TCGA data (n = 695).


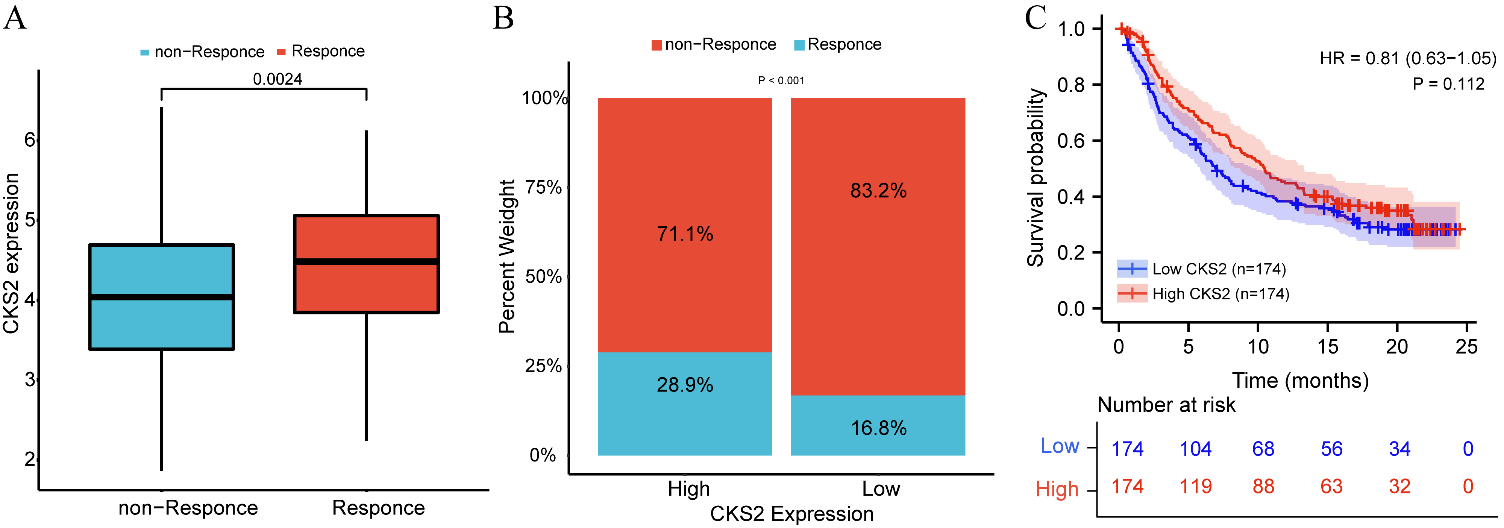


**Supplementary Figure 2. CKS2 in immune checkpoint inhibitors (ICIs).** (**A-C**) The Imvigor210CoreBiologies dataset was used to explore the efficacy of CKS2 expression in ICI prognostic markers; (**A**) Comparison of CKS2 between different immune responses; (**B**) Comparison of immune response rate between high expression group and low expression group CKS2; (**C**) Overall survival (OS) analysis of high-risk and low-risk populations.

**TABLE S1** Demographic and clinical characteristics of glioma patients with low- and high expression CKS2 in CGGA (n=811)

| **Characteristic** | **levels** | **CKS2 expression** | | ***p*** |
| --- | --- | --- | --- | --- |
|  |  | **Low**  **(n=406)** | **High**  **(n=405)** |  |
|  |  |  |  |  |
| Gender (%) | Female | 160 (39.4%) | 230 (56.8%) | 0.272 |
|  | Male | 246 (60.6%) | 175 (43.2%) |  |
| Age (meidan [IQR]) |  | 42 (11, 70) | 43 (8, 79) | < 0.001 |
| WHO grade | G2 | 190 (46.8%) | 51 (12.6%) | < 0.001 |
|  | G3 | 128 (31.5%) | 130 (32.1%) |  |
|  | G4 | 88 (21.7%) | 224(55.3%) |  |
| IDH status | WT | 142 (35.0%) | 218 (53.8%) | < 0.001 |
|  | Mut | 264 (65.0%) | 187 (46.2%) |  |
| 1p/19q codeletion | codel | 108 (26.6%) | 65 (16.0%) | < 0.001 |
|  | non-codel | 298 (73.4%) | 340 (84.0%) |  |

CGGA, Chinese Glioma Genome Atlas; CKS2, Cyclin-dependent kinase regulatory subunit 2

**TABLE S2** CKS2 interference sequence.

| Interfering sequence name | The target sequence |
| --- | --- |
| CKS2_001 | GACGACCTCTTCCAAAAGA |
| CKS2_002 | TCGACGAACACTACGAGTA |
| CKS2_003 | GTCTAGGCTGGGTTCATTA |
| CKS2_004 | CCTGTGCATGAGCTGTATT |
| CKS2_005 | CTGCAAGTAGGTTACTGTA |
